# Menthol-Based Hydrophobic Eutectic Solvents as Green Plasticizers for Biobased Acrylic Polymers

**DOI:** 10.1021/acsapm.5c03749

**Published:** 2025-12-24

**Authors:** Janire Aramberri, Matías L. Picchio, Aitor Barquero

**Affiliations:** † POLYMAT and Department of Applied Chemistry, University of the Basque Country UPV/EHU, Joxe Mari Korta Center, Tolosa Hiribidea, 72, 20018 Donostia, Spain; ‡ POLYMAT, Department of Mining-Metallurgy Engineering and Materials Science, School of Engineering, University of the Basque Country (UPV/EHU), Plaza Torres Quevedo 1, 48013 Bilbao, Spain; § IKERBASQUE, Basque Foundation for Science, Plaza Euskadi 5, 48009 Bilbao, Spain

**Keywords:** menthol-based HES, IBOMA, 2-OMA, biobased monomers, green plasticizers

## Abstract

While the polymer industry has gained importance in the last decades, many related environmental issues have appeared, caused by oil-based monomers, unsustainable polymerization processes, and toxic additives, among others. For instance, plasticizers commonly used in acrylic polymers often qualify as volatile organic compounds (VOCs), raising concerns due to their contribution to indoor air pollution, regulatory restrictions, and potential health risks. In this work, we explore a series of menthol-based hydrophobic eutectic solvents (HESs) as green alternatives to conventional organic plasticizers for acrylic films based on biobased isobornyl methacrylate (IBOMA), 2-octyl methacrylate (2-OMA), and their copolymers synthesized via miniemulsion polymerization. The biobased films were thoroughly characterized to assess their chemical structure, thermal, adhesive, and mechanical properties and their performance in a potential application as therapeutic patches, leveraging the inherent bioactive properties, of the HESs. We have found that the plasticizer effect on these biobased polymers could be controlled by the polarity of the hydrogen bond donor and the vapor pressure of the individual components in the eutectic mixture. These findings highlight the potential of menthol-based HESs as multifunctional, sustainable plasticizers, paving the way for environmentally friendly alternatives in advanced acrylic materials.

## Graphical abstract

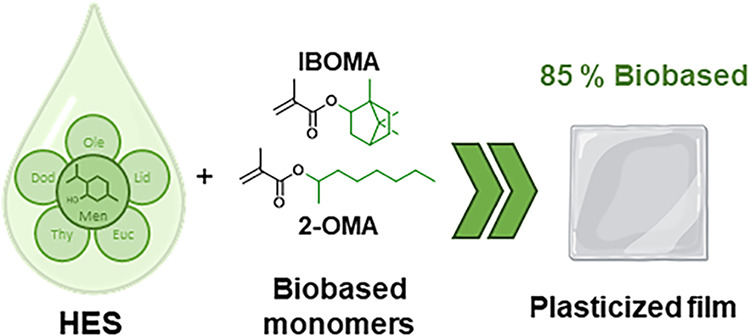

## 1 Introduction

The widespread use of polymeric materials in modern society has brought undeniable benefits in terms of versatility, cost, and performance.^1–3^ However, the environmental and health impacts associated with conventional polymer production have become increasingly difficult to ignore.^4–6^ Among the most pressing concerns are the reliance on petroleum-derived monomers, energy-intensive and unsustainable polymerization processes, and the incorporation of toxic additives such as volatile organic compound (VOC) plasticizers.^7–9^ These issues have spurred a growing interest in the development of more sustainable, safer alternatives across the polymer value chain.^10,11^

Acrylic polymers, widely used in coatings, adhesives, and films, are no exception. They often require the addition of plasticizers to achieve desirable mechanical properties and flexibility by lowering their glass transition temperature (*T*_g_).^12,13^ Unfortunately, many commonly used plasticizers, including phthalates and other low-molecular-weight compounds, are VOCs. These substances can volatilize during application and use, contributing to indoor air pollution, regulatory limitations, and potential adverse health effects.^14,15^ Consequently, there is a strong need for nontoxic, low-volatility plasticizers that are compatible with acrylic matrices and derived from renewable resources.^9,16,17^

In recent years, deep eutectic solvents (DESs) have emerged as promising green solvents due to their tunable properties, low toxicity, and ease of preparation from biobased components.^18–20^ DESs are defined as eutectic solvents whose components present enthalpic-driven negative deviations from thermodynamic ideality.^21,22^ In particular, hydrophobic eutectic solvents (HESs) composed of nonpolar or slightly polar molecules, such as those based on terpenes and monocarboxylic acids, are especially attractive due to their low water solubility; however, these systems typically exhibit only minor deviations from ideality and lack the significant melting point depression characteristic of DESs.^23^ Nonetheless, these mixtures typically remain in the liquid state at room temperature across a broad range of compositions.^24^ HESs have shown particular potential as green reaction media for biocatalytic processes, extraction media for pollutant removal, and functional additives in polymeric systems.^25–28^ HESs formed with natural compounds could offer an attractive route for sustainable material development, combining the plasticizing ability and additional functional properties with low toxicity. For instance,^29,30^ recent studies have explored the use of HESs as plasticizers in food packaging films based on low-density poly­(ethylene) or gelatin. However, limited efforts have been made to investigate their potential in other polymeric matrices, particularly those based on renewable resources.

In this work, we investigate the use of menthol-based HESs (Table 1) as green plasticizers in acrylic films synthesized via miniemulsion polymerization using biobased isobornyl methacrylate (IBOMA, 71% renewable content based on carbons), 2-octyl methacrylate (2-OMA, IBOMA, 67% renewable content based on carbons), and their copolymers. The two monomers were selected due to their commercial availability and relatively high biobased content. By exploring how the nature of the HES components affects their plasticizing behavior, we aim to identify environmentally friendly alternatives to conventional VOC plasticizers. HESs based on long-chain fatty acids exhibited the most effective plasticizing behavior and were therefore selected for direct incorporation during the miniemulsion polymerization process, resulting in homogeneous, high-quality acrylic films. Given the bioactive properties of the HESs explored, these biobased films hold promise for applications in the biomedical field.

**Table 1:**
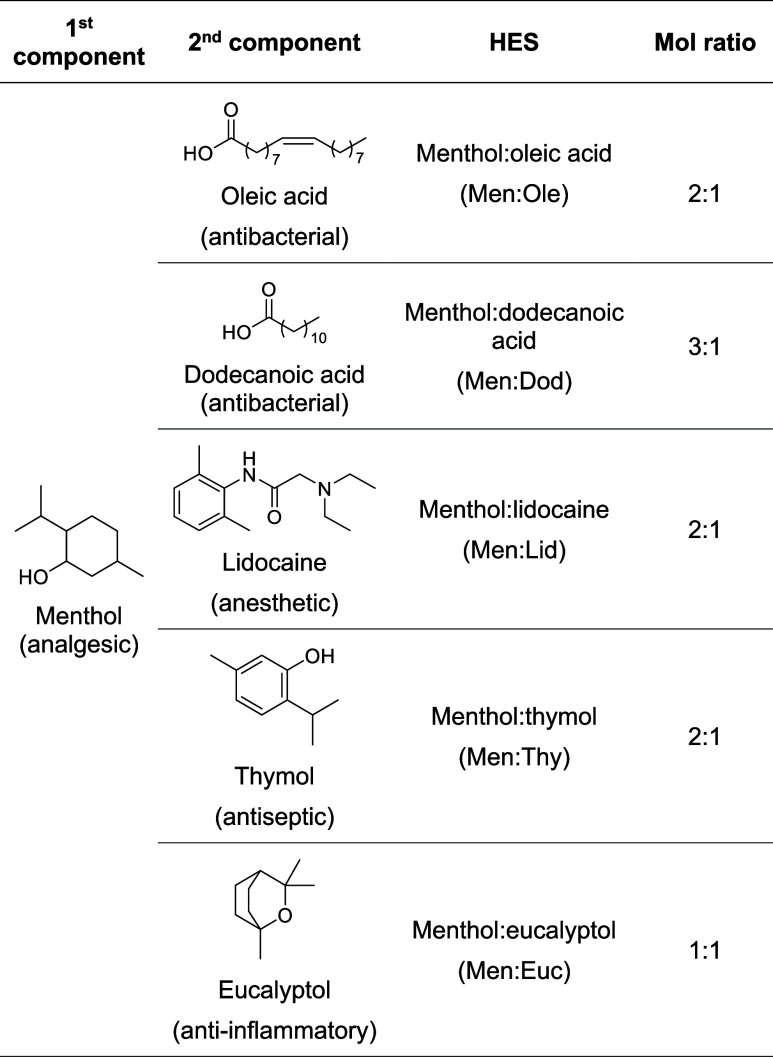
Chemical Structures, Mol Ratios, and Therapeutic Properties of the Components of the HESs^31–36^

Overall, this study contributes to broader efforts to replace hazardous additives in polymers, paving the way for more sustainable, functional acrylic materials.

## 2 Experimental Section

### 2.1 Materials

Dodecanoic acid (Dod), lidocaine (Lid), eucalyptol (Euc), and potassium persulfate (KPS) were all purchased from Sigma-Aldrich. Menthol (Men), oleic acid (Ole), thymol (Thy), cyclohexane, and sodium chloride were obtained from Thermo Fisher Scientific. Hydroquinone and magnesium sulfate (MgSO_4_) were acquired from Across Organics, and sodium bicarbonate (NaHCO_3_) was from Panreac. Isobornyl methacrylate (IBOMA, VISIOMER Terra IBOMA) and 2-octyl methacrylate (2-OMA, VISIOMER Terra OCMA) were kindly provided by Evonik Industries, and Dowfax 2A1 surfactant was supplied by Dow Chemicals.

For analytical purposes, deuterated chloroform (CDCl_3_) and deuterium oxide (D_2_O) for NMR were purchased from Eurisotop, while HPLC-grade tetrahydrofuran (THF) was obtained from Scharlab. Deionized water was used in all of the reactions. All chemicals were used as received without further purification.

### 2.2 Preparation of the Hydrophobic Eutectic Solvents (HESs)

Five HESs were prepared at the molar ratios as presented in Table 1.^37–39^ First, menthol was heated at 50 °C, and the second compound was dissolved under mechanical stirring until a transparent liquid was obtained. After cooling, the HES remained liquid in all cases indefinitely.

### 2.3 Synthesis of the Polymers

Two polymers were used in the study: a high-*T*_g_ IBOMA homopolymer (≈150 °C) and a lower-*T*_g_ IBOMA/2-OMA copolymer (57.6/42.4 weight ratio, ≈70 °C). Both latexes were synthesized through batch miniemulsion polymerization, with a target solid content of 20%. The detailed formulation for each system is shown in Table S1. The miniemulsions were produced by sonicating the organic and aqueous phase mixtures in a Branson Digital Sonifier SFX250 immersed in an ice bath to operate at 70% amplitude and 0.5 s pulses. After the addition of the initiator (KPS), the resulting miniemulsions were distributed into 100 mL bottles and sealed. The bottles were then placed in a 70 °C water bath and rotated end-overend at 49 rpm for 4 h. Finally, the obtained latexes were allowed to cool to room temperature before storage. Figure 1 shows the reaction scheme for the homopolymerization of IBOMA (A) and the copolymerization of IBOMA and 2-OMA (B).

**Figure 1:**
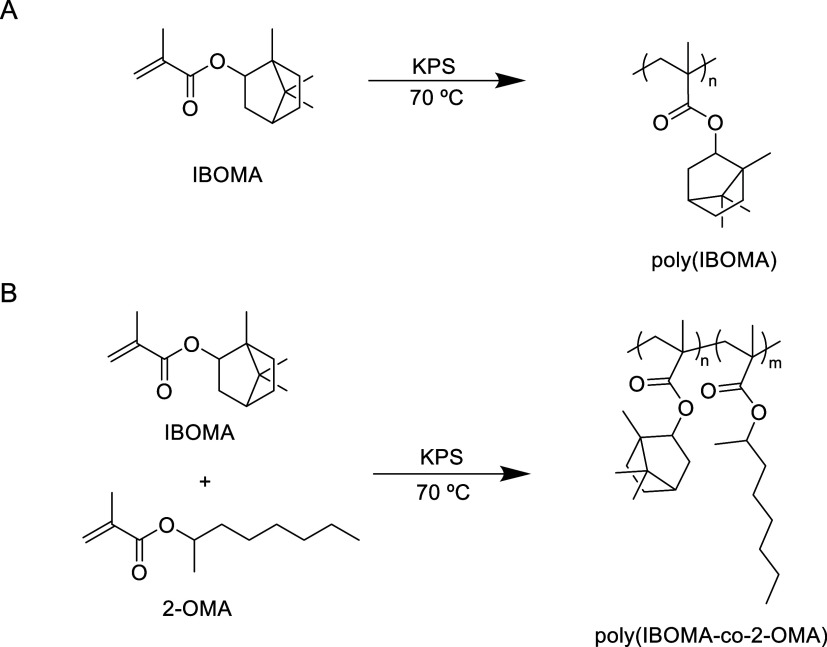
Polymerization scheme for the homopolymerization of IBOMA (A) and the copolymerization of IBOMA and 2-OMA (B).

Lastly, the latexes were characterized. The results are shown in Table S3.

### 2.4 Preparation of the Plasticized Biobased Polymer Films

The latexes were dried at room temperature in aluminum capsules for 24 h to obtain the dry polymer. This polymer was then dissolved in THF at a concentration of 300 mg/g, and the HES was added.^29^ Polymer:HES weight ratios of 80:20, 70:30, 60:40, 50:50, and 40:60 were tested. The resulting mixtures were cast into aluminum capsules and dried at 23 ± 3 °C and 50 ± 5% relative humidity for approximately 2.5 h. The drying time was established through weight-loss monitoring, as shown in Figure S5. After being dried, the films were demolded and stored in sealed bags to prevent HES evaporation.

Following an initial screening, the optimal polymer:HES weight ratios were identified based on the ability to form self-standing films with moderate tackiness. For the IBOMA homopolymer, a 50:50 ratio was selected, while for the IBOMA/2-OMA copolymer, the optimal ratio was 70:30. The other compositions resulted in materials that were either too brittle or excessively soft and tacky.

### 2.5 Synthesis of Latex Materials Incorporating HESs

As the material preparation method involved the use of THF as a cosolvent, we attempted two methods to directly incorporate HES into the latex. Unfortunately, when the HES was added to the preformed latex, coagulation and phase separation were immediately produced. Alternatively, we explored the inclusion of the HES in the organic phase for the miniemulsion polymerization process. Men:Ole HES was chosen for this strategy, and the latex formulation is shown in Table S2.

In this case, in addition to sonication (using the same settings), high-pressure homogenization was required to achieve smaller and more uniform monomer droplet sizes. After 10–12 homogenization cycles, the miniemulsion polymerizations were carried out in a 500 mL jacketed glass reactor equipped with a mechanical turbine stirrer, condenser, and nitrogen inlet. The reactor temperature was controlled by a thermostatic bath, and upon reaching 70 °C, an aqueous solution of KPS was added as an initiator. The reactions proceeded for 4 h, and the obtained latexes were allowed to cool to room temperature before storage. Finally, they were characterized, obtaining the results shown in Table S3.

### 2.6 Characterization

#### 2.6.1 Fourier Transform Infrared spectroscopy (FTIR)

Infrared spectra were obtained on an α II Compact FTIR spectrometer after performing 10 scans at a resolution of 4 cm^–1^.

#### 2.6.2 Nuclear Magnetic Resonance Spectroscopy (NMR)

^1^H NMR measurements were performed by using a Bruker AVANCE 400 MHz spectrometer. For sample preparation, aqueous samples were mixed with D_2_O (400 μL of water with 50 μL of D_2_O), and organic samples were prepared by mixing 50 μL of the sample with 400 μL of CDCl_3_.

#### 2.6.3 Dynamic Light Scattering (DLS)

The *Z*-average particle diameters of the latexes were determined using a Zetasizer Nano ZS equipped with Zetasizer software. Samples were prepared by diluting a drop of latex in Milli-Q water. Measurements were conducted at 20 °C following a one min temperature equilibration and consisted of three consecutive size measurements. The reported value represents the average of the three measurements.

#### 2.6.4 Thermogravimetric Analysis (TGA)

A TA Instruments Q500 equipment was used to measure the weight loss of the films and pure HESs. The measurements were done under a nitrogen atmosphere from 25 to 800 °C at a heating rate of 10 °C/min.

#### 2.6.5 Differential Scanning Calorimetry (DSC)

DSC measurements were performed using a DSC Q2000 instrument coupled with TA Universal Analysis software. Samples (3–10 mg) were sealed in hermetic aluminum pans and subjected to heating–cooling–heating cycles between −80 and 80 °C at a rate of 20 °C/min.

#### 2.6.6 Tensile Test

Tensile tests were conducted using a TA.HDplus Texture Analyzer equipped with a 30,000 g load cell and controlled via Exponent software. Bone shape specimens with standardized dimensions were cut, and tests were performed at a constant crosshead speed of 0.42 cm/s in a room with controlled temperature (23 ± 5 °C) and relative humidity (50 ± 5%).

#### 2.6.7 Probe Tack Test

A TA.HDplus Texture Analyzer equipped with Exponent software and a 5 mm diameter stainless steel cylindrical probe was used for the measurements. Films were cast onto glass substrates with a uniform thickness of 0.1 mm and dried at 23 ± 5 °C and 50 ± 5% relative humidity. Referring to the test, a force of 4.5 N was applied for 1 s of contact between the probe and the film.

#### 2.6.8 Water Uptake

For water uptake measurements, each film was immersed in water for 7 days and weight changes were monitored at regular intervals. Prior to being weighed, the film surfaces were gently blotted with tissue paper to remove excess water.

#### 2.6.9 Contact Angle

Contact angle measurements were performed using a Contact Angle System, a Model OCA equipped with SCA20 software. Films with a thickness of 0.1 mm were dried on glass substrates at 23 ± 5 °C and 50 ± 5% R.H. An average calculated from 10 droplets is reported.

#### 2.6.10 Rheological Tests

Rheological measurements were performed using an Anton Paar MCR 101 torsional rheometer equipped with an 8 mm diameter plate–plate geometry and operated with RheoPlus software. Strain sweep was carried out at 25 °C by varying the strain amplitude from 0.1% to 100% at a fixed frequency of one Hz. Frequency sweep was conducted at the same temperature, ranging from 0.1 to 100 s^–1^ with a constant strain amplitude of 0.1%. Temperature sweep was performed from 0 to 80 °C at a fixed strain amplitude of 0.1% and a frequency of 1 Hz.

#### 2.6.11 Drying Experiments

The drying process of the films and the evaporation of the HESs over time were monitored by using a microbalance connected to the Camile TG software at room temperature.

#### 2.6.12 Minimum Film-Forming Temperature (MFFT)

For the latex samples synthesized with HES in the organic phase, the MFFT was measured in RHO-MFFT90 equipment. Films were applied on top of aluminum foil using a film applicator with a thickness of 250 μm, and they were dried with an airflow of 4 L/min at a pressure of 4 bar. The temperature ranges were 15–33 and 5–23 °C for the poly­(IBOMA): Men:Ole and poly­(IBOMA-*co*-2-OMA): Men:Ole latexes, respectively.

#### 2.6.13 Gravimetric Analysis

The solid content (S.C.) and conversion (*x*) of the latexes were calculated by drying a sample in a ventilated oven at 65 °C until a constant weight was achieved. Then, eqs 1 and 2 were used for these calculations, respectively.

1 $$S.C.=\frac{\mathrm{weight}\;\mathrm{of}\;\mathrm{the}\;\mathrm{dry}\;\mathrm{sample}}{\mathrm{weight}\;\mathrm{of}\;\mathrm{the}\;\mathrm{latex}\;\mathrm{sample}}$$

2 $$x=\frac{S.C.\cdot L_{T}-I-S}{M_{0}}$$

*L*_T_ is the total weight of the synthesized latex (g), *I* and *S* are the amount of initiator and surfactant added (g), and *M*_0_ is the initial monomer amount (g).

#### 2.6.14 Gel Permeation Chromatography (GPC)

GPC was performed by using a system comprising a Shimadzu LC-20A pump, a Waters 717 autosampler, a Waters 2410 differential refractometer detector, and Styragel HR2, HR4, and HR6 columns. Measurements were carried out at 35 °C by using HPLC-grade THF as the mobile phase at a flow rate of 1 mL/min. Molecular weight distributions were determined relative to those of polystyrene standards.

Samples were prepared by drying the latexes at room temperature and dissolving them in THF to a concentration of 3 mg/mL. Prior to injection, the solutions were filtered through a 0.45 μm pore size filter, and a droplet of toluene was added to each solution as an internal reference.

## 3 Results and Discussion

### 3.1 Characterization of the HESs

FTIR and ^1^H NMR were used to analyze the HESs’ chemical structure (Figures S1 and S2). Both techniques confirmed that the HESs retained the spectral features of their original components with no evidence of new covalent bond formation. Additionally, evidence of hydrogen bonding can be observed in the FTIR spectra, particularly for Men:Ole and Men:Dod (Figure S1A,B, respectively). In these cases, the characteristic carbonyl stretching band, typically located around 1710 cm^–1^, shifts to higher wavenumbers in the HES spectra compared with the spectra of the pure fatty acids, suggesting the involvement of the carbonyl group in hydrogen bonding interactions.

Drying experiments were performed to evaluate the volatility of the HESs at room temperature and atmospheric pressure (Figure 2A). Evaporation rates, determined from the slopes of the linear regions of the drying curves, are presented in Figure 2B.

**Figure 2:**
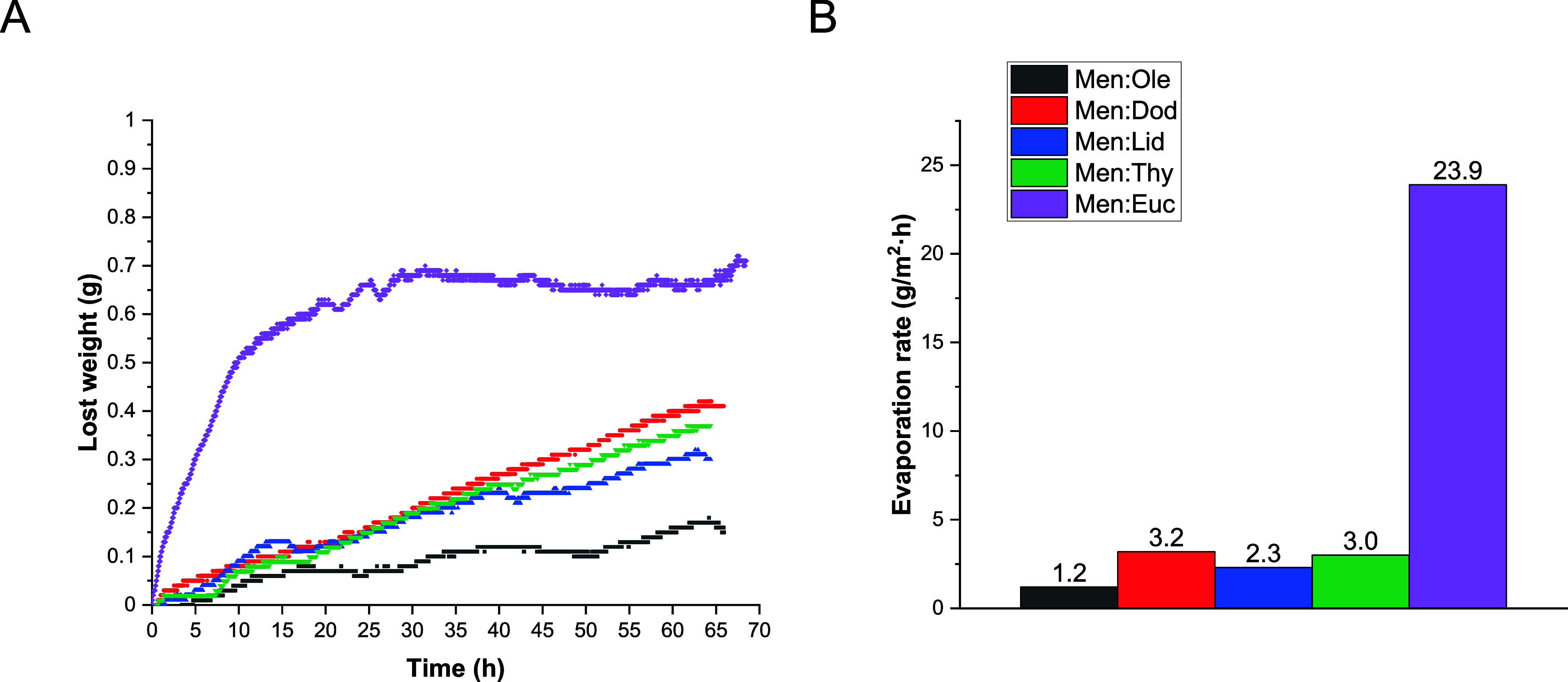
(A) Weight-loss curves over time of the HESs obtained at 25 °C and 1 atm. (B) Evaporation rate of each HES obtained from (A).

Although four of the HESs exhibit similar overall volatility, Men:Ole remains in the liquid state for a longer period, whereas Men:Euc shows the highest volatility. This property significantly influences the performance of the resulting materials as a faster evaporation rate leads to a shorter plasticizing effect, resulting in an increased rigidity of the polymer matrix. For this reason, although all HESs were used to synthesize and evaluate materials, the two extreme cases, Men:Ole and Men:Euc, were selected for a more detailed study.

To better understand their evaporation behavior, ^1^H NMR spectroscopy was used to determine whether both components of each HES evaporate simultaneously or if they evaporate independently. Samples were collected after 48 h of evaporation and analyzed for their molar composition. The results show that most HESs maintain a constant molar ratio during evaporation (Table 2), indicating azeotrope-like behavior. In contrast, the Men:Euc mixture shows a change in composition over time, indicating that its components evaporate at different rates. After 48 h, only solid menthol was detected in the sample, suggesting that the high overall volatility of the mixture is largely driven by the high vapor pressure of eucalyptol.

**Table 2:** Composition of the HES at the Moment of Preparation (*t* = 0) and after 48 h of Evaporation

HES	Men:Ole	Men:Dod	Men:Lid	Men:Thy	Men:Euc
composition at *t* = 0 (mol:mol)	2.0:1.1	3.0:1.1	2.0:1.0	2.0:1.0	1.0:1.1
composition at *t* = 48 h (mol:mol)	2.0:1.4	3.0:1.1	2.0:1.2	2.0:1.0	1.0:0.0

### 3.2 Physicochemical Properties of the HES-Plasticized Acrylic Films

A total of ten films were synthesized, with five based on poly­(IBOMA) and five based on the poly­(IBOMA-*co*-2-OMA) copolymer (IBOMA/2-OMA), each formulated with a different HES as a plasticizer. The characterization results of the latexes used for the film preparation are presented in Section S2.

FTIR analysis was conducted to investigate the nature of interactions between the polymer matrix and HES in the plasticized films. No significant changes were observed in the spectra upon comparison of the films to the individual components, discarding hydrogen bonding effects between the HES and the polymer. Therefore, the plasticization occurs through physical interactions, such as van der Waals forces, which promote increased polymer chain mobility by disrupting intermolecular forces within the network and lower the *T*_g_. The film containing Men:Ole is given as an example in Figure 3A, but the complete set of FTIR spectra is provided in Figure S6 for the homopolymer films and in Figure S7 for the copolymer films.

**Figure 3:**
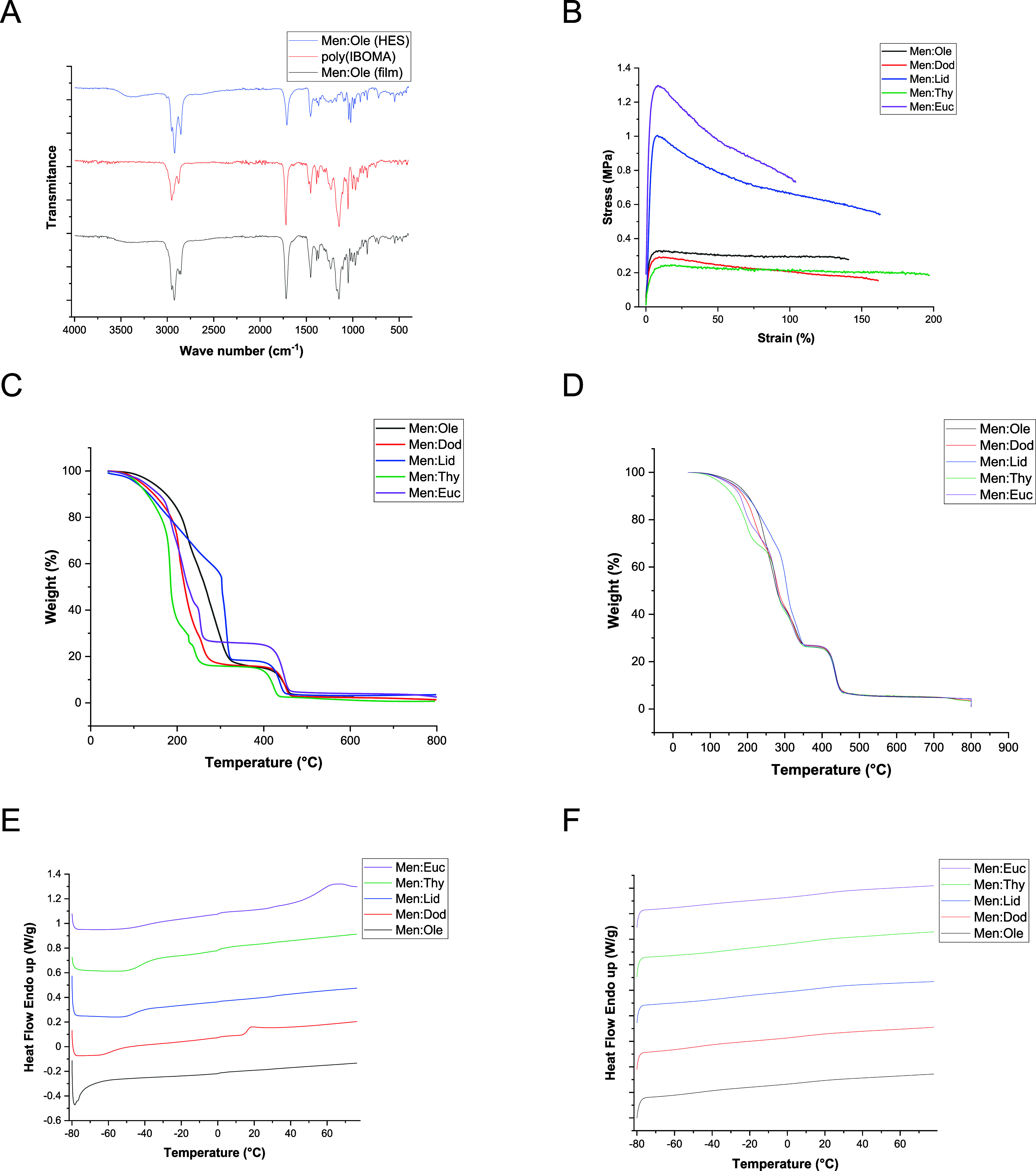
Physicochemical properties of the HES-plasticized acrylic films: (A) FTIR spectra of the neat IBOMA homopolymer and HES compared to the plasticized film. (B) Stress vs strain curves of the copolymer films. (C) TGA curves of the plasticized homopolymer films. (D) TGA curves of the plasticized copolymer films. (E) DSC curves of the plasticized homopolymer films. (F) DSC curves of the plasticized copolymer films.

Tensile tests were carried out to evaluate the mechanical performance of the plasticized films. These tests were conducted exclusively on the materials containing the copolymer, as they exhibited a more ductile behavior during handling compared with the homopolymer-based films, which were too brittle for reliable testing. The results are presented in Figure 3B.

The results showed that all of the films were soft and exhibited good elasticity, consistent with effective plasticization. In contrast, the film containing Men:Euc was relatively brittle, displaying the highest tensile strength (1.3 MPa) but lower strain at break (100%), suggesting limited chain mobility due to rapid plasticizer loss. On the contrary, the materials containing Men:Ole, Men:Dod, and Men:Thy showed the lowest tensile strength (less than 0.4 MPa) and higher elongations at break, between 140 and 200%. The material plasticized with Men:Lid exhibited intermediate mechanical behavior, bridging the properties of the more elastic and brittle formulations with a relatively high tensile strength at 1 MPa, but still with an elongation at break of more than 150%, showing the best promise as a plasticizer for the acrylic copolymer.

TGA and DSC analyses were carried out to investigate the thermal properties of the plasticized films. TGA results are presented in Figure 3C,D for the homopolymer- and copolymer-based films, respectively. Key thermal degradation parameters are summarized in Table 3, including the temperatures at 5% (*T*_5%_), 50% (*T*_50%_), and maximum (*T*_max_) weight loss.

**Table 3:** Thermal Degradation Parameters of the Plasticized Films Obtained by TGA

homopolymer				copolymer			
film	*T*_5%_ (°C)	*T*_50%_ (°C)	*T*_max_ (°C)	film	*T*_5%_ (°C)	*T*_50%_ (°C)	*T*_max_ (°C)
Men:Ole	142.0	264.8	288.4	Men:Ole	169.8	278.7	433.8
Men:Dod	116.3	217.2	205.8	Men:Dod	159.0	283.4	273.6
Men:Lid	104.5	303.5	303.0	Men:Lid	161.1	306.8	303.5
Men:Thy	107.6	185.4	226.2	Men:Thy	132.7	280.5	277.4
Men:Euc	123.6	225.5	252.5	Men:Euc	152.8	280.7	273.6

These thermal degradation results can be qualitatively compared to both the evaporation behavior of the pure HESs at room temperature (Figure 2A) and their individual thermal degradation profiles (Figure S3A). In the case of the pure HESs, a consistent trend is observed between volatility under ambient conditions and TGA results; Men:Ole shows the lowest volatility and highest thermal stability, while Men:Euc is the most volatile.

However, when they are incorporated into the polymer matrices, this trend is no longer maintained, particularly in the copolymer films. While homopolymer-based films generally show earlier degradation due to their higher HES content, the relative thermal stability of the different HES-plasticized films shifts notably.

For example, in the copolymer series, the film containing Men:Ole stands out as the most thermally stable, with a *T*_5%_ of 169.8 °C and a *T*_max_ of 433.8 °C, suggesting strong interactions between this HES and the polymer matrix. In contrast, Men:Thy, despite being less volatile as a pure component than Men:Euc, shows a lower *T*_5%_ (132.7 °C), indicating an earlier onset of degradation in the film form.

These discrepancies show that the thermal behavior of the plasticized materials is governed not only by the intrinsic volatility or degradation temperature of the pure HESs, but also by the nature and strength of the physical interactions between each HES and the polymer network. Note that, as presented in Figure S4A, the neat polymers show similar transitions to the plasticized films. These interactions may affect how tightly the plasticizer is retained within the film and how it responds to thermal stress.

DSC analysis was performed to determine the *T*_g_ of each material by calculating the first derivatives of the heat flow curves shown in Figure 3E,F. The glass transition temperatures obtained from these analyses are summarized in Table 4.

**Table 4:** *T*_g_ Values of the Polymer Films Were Calculated from DSC Analysis

*T*_g_ (°C)	polymer	Men:Ole	Men:Dod	Men:Lid	Men:Thy	Men:Euc
homopolymer	150	0	15	0	0	30
copolymer	72^a^	0	5	0	0	20

a Measured by DSC of the pure copolymer (see Figure S4B).

Since all the films exhibited a soft and flexible behavior, *T*_g_ values were determined solely on the basis of the thermal transitions occurring near room temperature. An exception was observed in the films containing Men:Euc, which were more brittle, likely due to partial loss of the plasticizer prior to analysis, as Men:Euc is the most volatile of the five HESs. Additional transitions observed in the DSC curves may correspond to thermal events associated with the HESs themselves, as suggested by a comparison with the DSC profiles of the pure liquids (Figure S3B).

Additionally, rheological measurements were also performed on all materials to further analyze their viscoelastic behavior. Figure 4 presents the frequency and temperature sweeps for the homopolymer (A and C) and copolymer (B and D) for the materials containing Men:Ole as a representative system.

**Figure 4:**
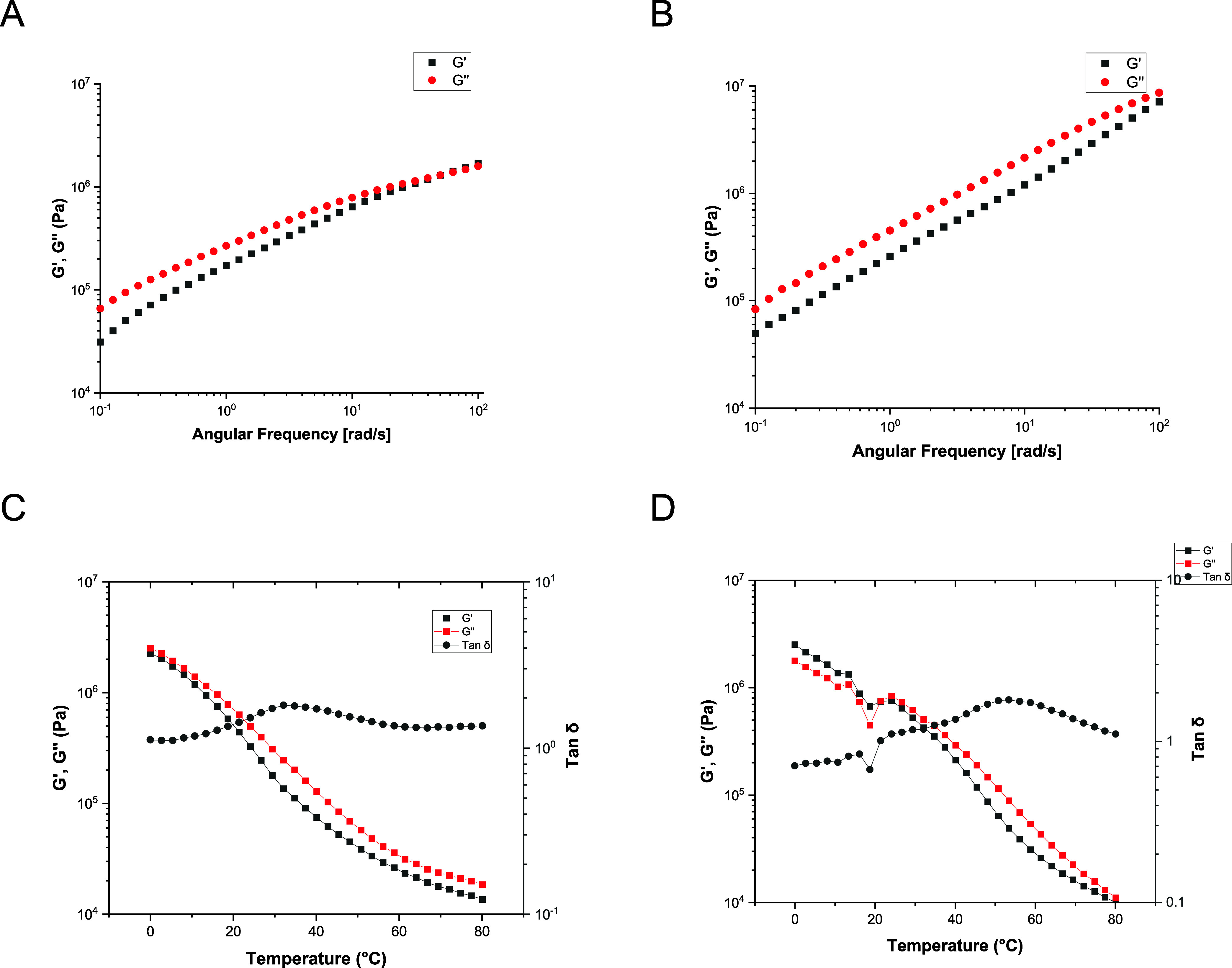
Rheological properties of the homopolymer and copolymer films plasticized with Men:Ole. Frequency sweeps for the homopolymer (A) and copolymer films (B). Temperature sweeps for the homopolymer (C) and copolymer films (D).

Frequency sweeps revealed that both the plasticized homopolymer and copolymer films show a viscoelastic liquid-like behavior at low to mid frequencies with *G*″ > *G*′. Plasticizers increase molecular mobility, making the polymer more compliant and allowing greater energy dissipation during deformation. This leads to a higher loss modulus, especially at low frequencies where viscous flow dominates.^40^ At low frequencies, polymer chains have more time to respond to deformation, favoring viscous motion over elastic recoil, which further elevates *G*″ above *G*′ in plasticized systems.^41^ A predominantly viscoelastic liquid behavior was observed throughout most of the temperature range explored in the temperature sweeps (0–80 °C). A maximum in tan δ, associated with *T*_g_, was identified at approximately 33 °C for the homopolymer and 53 °C for the copolymer films. Comparable trends were also observed for the other HES plasticizers, as shown in Figures S8 and S9.

#### 3.2.1 Potential of the HES-Plasticized Films as Therapeutic Patches

The combination of mechanical flexibility and bioactive composition makes HES-plasticized acrylic films promising candidates as therapeutic patches for wound care. The natural components of the HESs, terpenes and long-chain fatty acids, are known for their antimicrobial, anti-inflammatory, and skin-healing properties, which could offer added therapeutic benefits when incorporated into polymeric materials.

In this context, films plasticized with volatile HESs show particular potential, as their high volatility could be strategically exploited in wound healing applications requiring temporary adhesion. We observed that some HES-containing films initially exhibit high flexibility and strong adhesion, properties that are desirable for effective skin contact and protection. As the plasticizer gradually evaporates, the film loses its tackiness, potentially allowing for painless removal without disturbing the healing tissue. This ″on-demand″ adhesion behavior addresses a key limitation of conventional dressings and could offer a valuable advantage in the development of next-generation wound care materials.

To preliminarily evaluate this potential, a four day protocol combining tack testing and drying experiments was carried out, simulating the expected performance cycle of a therapeutic patch. This concept is illustrated schematically in Figure 5A.

**Figure 5:**
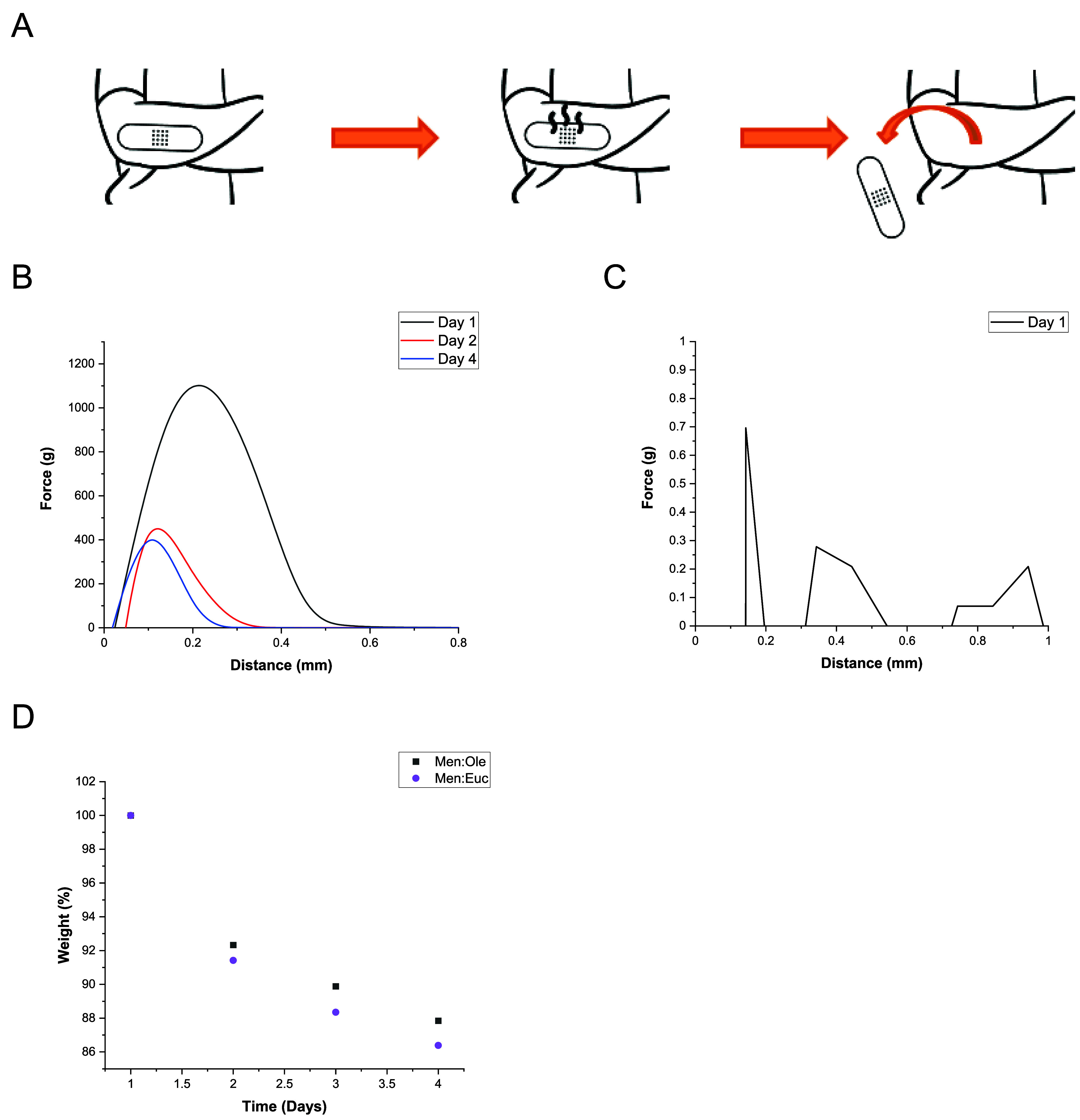
(A) Schematic of the performance of an ideal therapeutic patch. Adhesion force vs distance of the 60:40 copolymer film containing Men:Ole (B) and Men:Euc (C). (D) Weight loss of the films over time.

Two copolymer-based materials were prepared using Men:Euc, the most volatile plasticizer, and Men:Ole, the least volatile plasticizer, as reference points for comparison. A 60:40 polymer:HES ratio was selected to produce tackier films, enhancing the sensitivity of tack loss measurements over time. The results of the tack tests are shown in Figure 5B,C, while the corresponding weight-loss data are presented in Figure 5D. In this case, the initial weight on day one was taken as 100%, and all subsequent values were calculated relative to that baseline.

Interestingly, the material containing Men:Euc showed no noticeable tack even on the first day, suggesting that the plasticizer may have largely evaporated prior to testing. In contrast, the film with Men:Ole effectively retained its adhesive properties throughout the evaluation period, more closely aligning with the intended behavior for therapeutic patch applications. However, as indicated by the TGA results (Figure 3C,D), the difference in weight loss between the two films does not directly reflect the volatility observed in the pure HESs. This discrepancy likely arises from differences in the physical interactions between each HES and the polymer matrix. In this case, Men:Euc evaporated only slightly more than Men:Ole, despite its higher inherent volatility.

It is important to note that the initial HES content in both films at day one cannot be assumed to be equal, as some evaporation may have occurred during the THF removal step in the film preparation process. Therefore, the low tackiness observed in the Men:Euc film could be attributed to its higher evaporation rate, with plasticizer loss likely beginning before the measurements shown in Figure 5C were initiated.

In the context of their potential application as wound healing patches, another important property to consider is the hydrophilicity of the materials, as it can influence both comfort and interaction with exudates. To evaluate this, water uptake was monitored in the copolymer films over a period of 7 days (Figure 6A).

**Figure 6:**
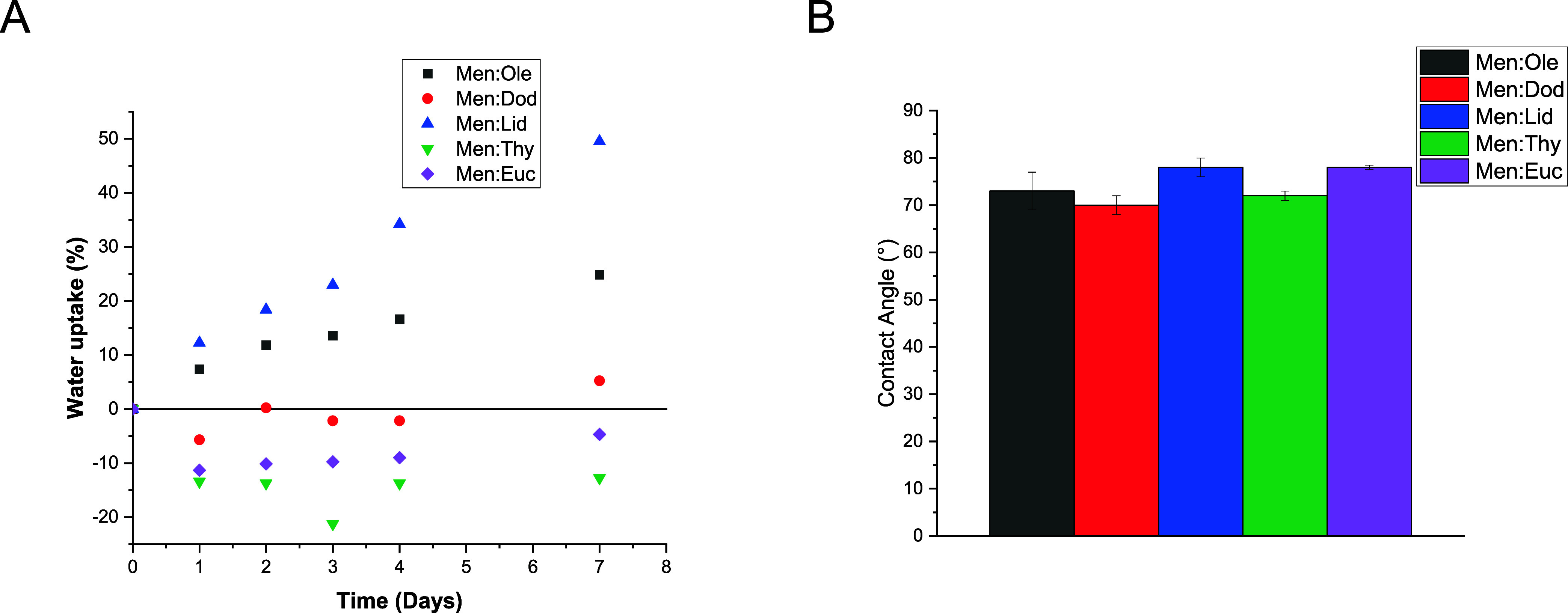
Water resistance properties of the plasticized copolymer films. (A) Water uptake over 7 days. (B) Contact angle with water.

The water uptake behavior was different depending on the material. In the case of Men:Ole and Men:Lid, a gradual increase of the weight is observed, indicating the absorption of water. For the films with Men:Dod, Men:Thy, and Men:Euc on the other hand, a negligible increase of weight or even a decrease was measured. All films developed a whitish appearance, indicating that water uptake occurred in all cases; therefore, in the case of Men:Dod, Men:Thy, and Men:Euc, the leaching of the HES surpassed the water uptake. To determine whether any structural or compositional changes took place, the immersion water of each film was analyzed by ^1^H NMR. The spectra are presented in Figure S10. The analysis confirmed that a portion of the HES had leached into the water, explaining the weight loss observed in some samples, while the copolymer matrix remained intact in the films.

In addition, the surface wettability of the films was evaluated by measuring the water contact angle. The mean values and corresponding standard deviations are summarized in Figure 6B.

Although the films exhibit some variability, they can all be broadly classified as hydrophobic. A comparison between water uptake and contact angle measurements reveals a general trend; films that absorbed more water also showed higher contact angles, while those with minimal weight change tended to be more hydrophilic. An exception to this pattern is the film containing Men:Euc. This behavior suggests that in certain cases, the HES may leach into the surrounding water and be replaced by absorbed water. As the HES contributes to the film’s hydrophobic character, its loss would lead to increased surface hydrophilicity.

### 3.3 Sustainable Preparation of the HES-Plasticized Acrylic Films

A clear limitation of the film preparation method developed is the use of THF as a cosolvent for mixing the HES and the preformed biobased acrylic polymer. To overcome this drawback and move toward more sustainable processes, two solvent-free approaches were explored.

The first approach involved the direct addition of the HES to the polymer latex, based on the hypothesis that the HES could swell the polymer particles and promote film formation upon drying (Figure 7A). As a preliminary test, Men:Ole was added to both homopolymer and copolymer latexes under stirring, targeting the final polymer: HES ratios of 50:50 and 70:30. However, the addition of the HES to the latex led to partial coagulation of the polymer (Figure 7I). After the coagulated fraction was filtered out, the remaining dispersion underwent phase separation during drying. As a result, the films formed were brittle and showed visible cracking, likely due to insufficient plasticization (Figure 7II,III).

**Figure 7:**
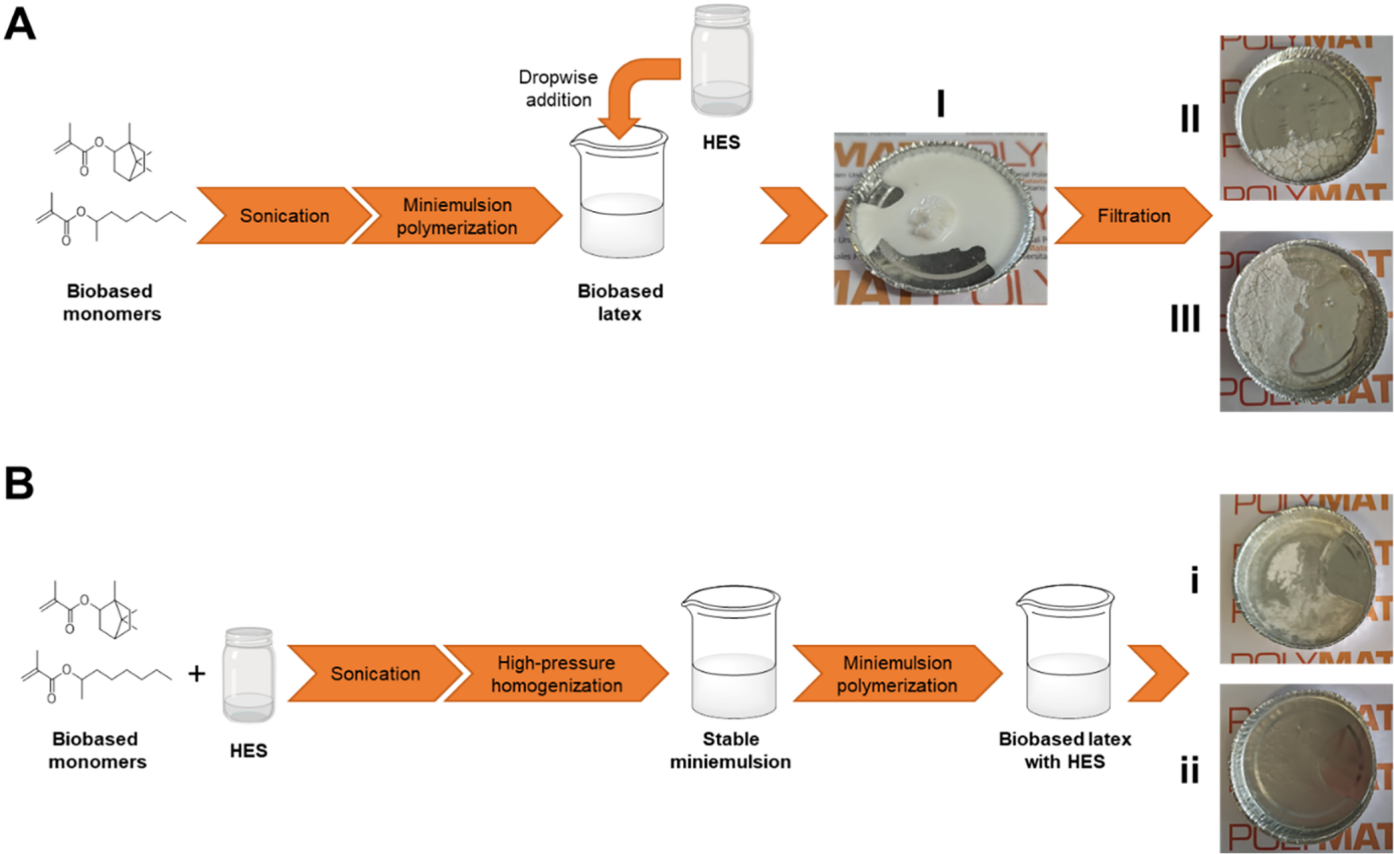
Schematic of both strategies. (A) addition of the HES to the latex, with coagulation ((I) and phase separation (II) for the homopolymer and (III) for the copolymer); (B) addition of the HES to the formulation and film formation of the latex ((i) for the homopolymer and (ii) for the copolymer).

The second strategy involved incorporating the HES directly into the polymerization process by including it in the initial miniemulsion formulation (Figure 7B). This method led to the successful synthesis of stable latexes in both systems, as the latexes remained stable for more than 14 months. Interestingly, although full conversion of the monomers was achieved, a decrease in the molar mass of the polymer was observed compared with the polymerization without HES (Table S3). The reduction in molar mass could be partially because the concentration of the monomers was decreased in the droplets, but it is also likely that some chain transfer reaction occurred. Moreover, as the HES used for this test was Men:Ole, some propagation to the double bonds in the oleic acid may also contribute to the reduction of the molar mass.

The film derived from the IBOMA homopolymer latex (Figure 7i) displayed some heterogeneity, whereas the copolymer-based film was visually homogeneous but slightly brittle (Figure 7ii). This brittleness may be due to the extended drying time required, which also resulted in the evaporation of a significant portion of the HES plasticizer.

To gain further insight into the film formation behavior, minimum film formation temperature (MFFT) tests were conducted. The homopolymer latex exhibited an MFFT close to 24 °C, while the copolymer latex showed a lower MFFT, around 9 °C. These findings help explain the slight opacity observed in the homopolymer film as limited chain mobility near room temperature likely hindered full particle coalescence.

Although the films prepared using this solvent-free strategy showed increased brittleness compared with the ones cast from THF, this issue could potentially be resolved by increasing the HES content in the initial formulation or by reducing the *T*_g_ of the biobased copolymer, to aid the film formation process at the same time. This approach provides a more sustainable alternative for producing biobased functional materials with improved environmental profiles.

## 4 Conclusions

This work presents a new class of biobased acrylic films plasticized with hydrophobic eutectic solvents (HESs), offering a sustainable alternative to conventional materials that rely on fossil-derived plasticizers. The resulting materials reached biobased contents between 78% and 85% and showed good film-forming capacity, tunable thermal and mechanical properties, and promising functional behavior.

Among the formulations studied, the film plasticized with Men:Ole showed the most suitable combination of flexibility, adhesion, and durability for potential use as a therapeutic patch. Its time-dependent loss of tackiness, driven by controlled evaporation of the plasticizer, highlights the possibility of developing ″on-demand″ adhesive materials for wound care applications. In contrast, highly volatile plasticizers such as Men:Euc led to premature tack loss, underlining the critical role of plasticizer–polymer interactions in defining performance.

Finally, a solvent-free synthesis strategy was successfully implemented by incorporating the HES directly into the polymerization process, yielding stable latexes capable of forming continuous films. This advancement paves the way for the more sustainable production of functional materials with high renewable content and targeted biomedical potential.

## Supplementary Material



## Notes

Characterization of the HESs. Synthesis and characterization of the polymers. Preparation of the HES-plasticized acrylic films. Physicochemical properties of the HES-plasticized acrylic films (PDF)
