# Supplementary material for: Menthol-Based Hydrophobic Eutectic Solvents as Green Plasticizers for Biobased Acrylic Polymers
Source: ACS Appl Polym Mater. 2025 Dec 24;8(1):350–60. doi: 10.1021/acsapm.5c03749 (PMC12797267; doi:10.1021/acsapm.5c03749)
Supplement: Supplementary file 1 [file ap5c03749_si_001.pdf]

Supporting Information

Menthol-based Hydrophobic Eutectic Solvents as Green Plasticizers for Biobased Acrylic Polymers

Janire Aramberri[1], Matías L. Picchio[2,3],* and Aitor Barquero[1]*

[1] POLYMAT and Department of Applied Chemistry, University of the Basque Country UPV/EHU, Joxe Mari Korta Center, Tolosa Hiribidea, 72, 20018 Donostia, Spain

[2] POLYMAT, Department of Mining-Metallurgy Engineering and Materials Science, School of Engineering, University of the Basque Country (UPV/ EHU), Plaza Torres Quevedo 1, 48013 Bilbao, Spain

[3] IKERBASQUE, Basque Foundation for Science, Plaza Euskadi 5, Bilbao, 48009 Spain

Corresponding authors:

matiasluis.picchiop@ehu.eus

aitor.barquero@ehu.eus

Outline

1. Characterization of the HESs 2
2. Synthesis and characterization of the polymers 4
   2.1. Synthesis of the latexes 4
   2.2. Characterization of the latexes 5
3. Preparation of the HES-plasticized acrylic films 6
4. Physicochemical properties of the HES-plasticized acrylic films 6
   4.1. Composition of the HES-plasticized acrylic films 6
   4.2. Rheological properties of the HES-plasticized acrylic films 8
   4.3. Potential of the HES-plasticized films as therapeutic patches 12

1. Characterization of the HESs

The chemical structure of the HESs was analyzed using FTIR and ^{1}H-NMR spectroscopy. The spectra of each HES were compared with those of their individual components to identify any shifts or changes in characteristic signals that might occur during preparation. FTIR spectra are presented in Figure S1 and the corresponding ^{1}H-NMR spectra are shown in Figure S2.

Figure **S3** shows the thermal properties of the pure HES, TGA (A) and DSC (B).

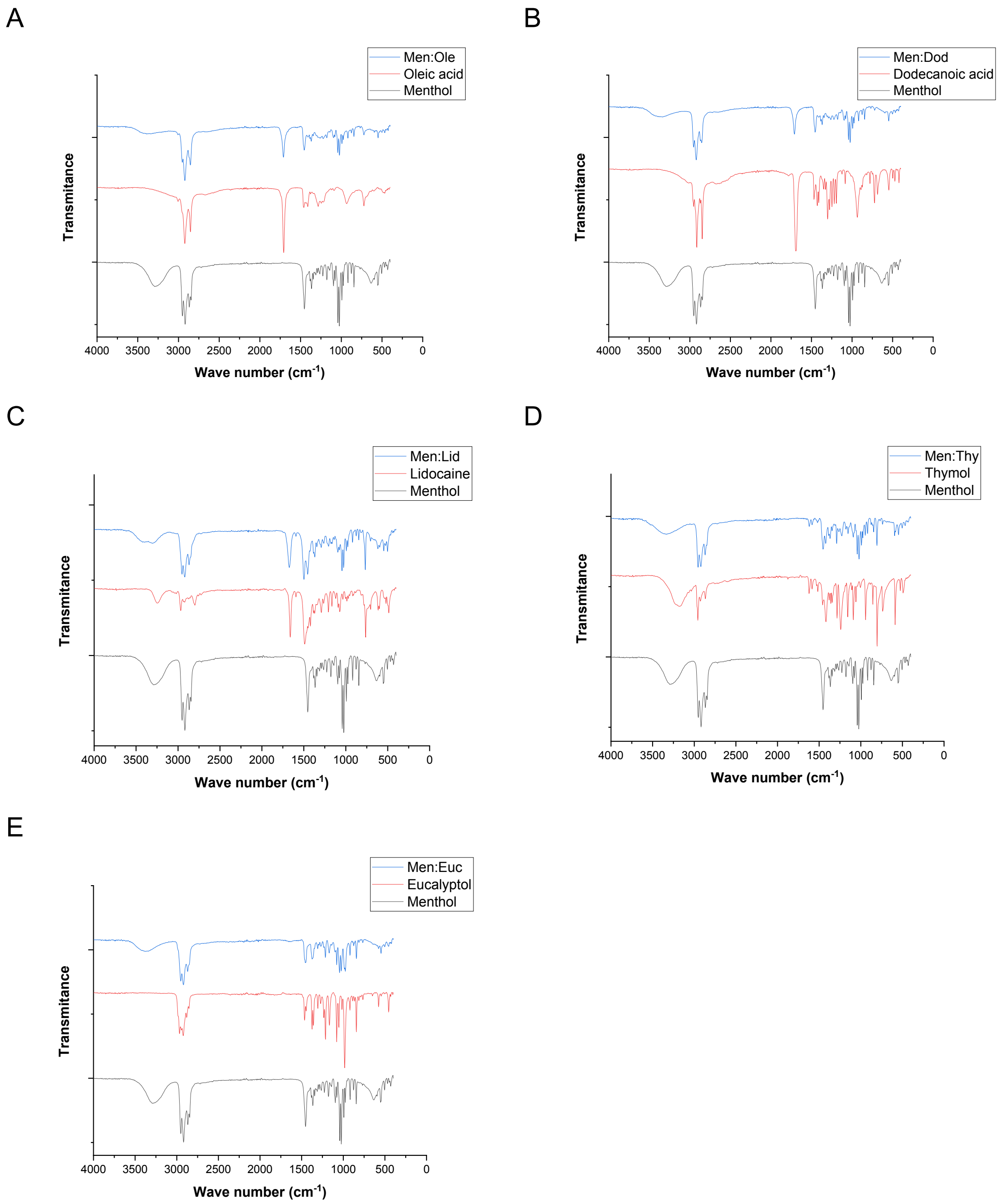

Figure S1. FTIR spectra of the HES and its components. In black the menthol, in red the second component and in blue the HES. A) Men:Ole, B) Men:Dod, C) Men:Lid, D) Men:Thy and E) Men:Euc.

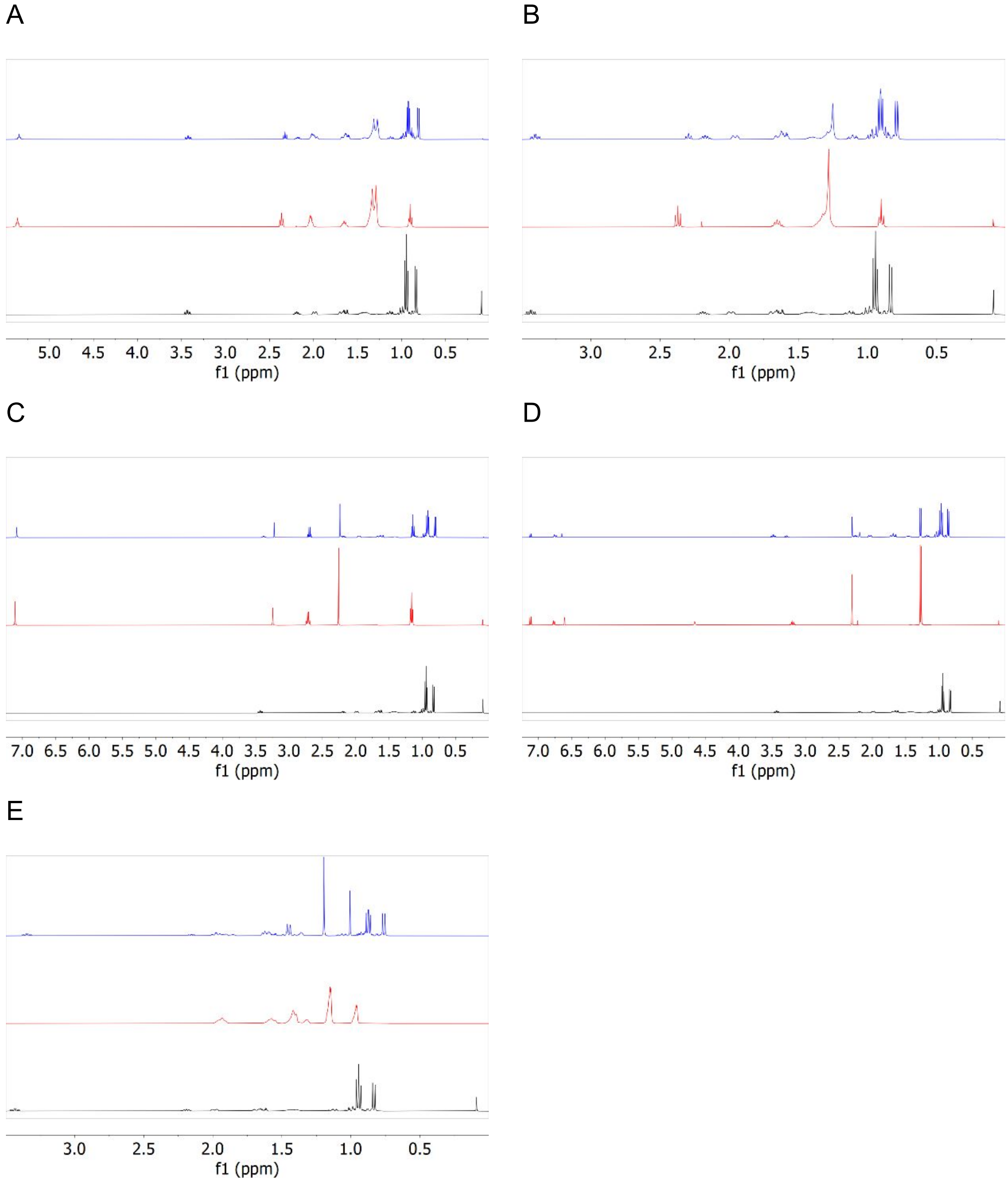

Figure S2. ^{1}H-NMR spectra of the HES and its components. In black the menthol, in red the second component and in blue the HES. A) Men:Ole, B) Men:Dod, C) Men:Lid, D) Men:Thy and E) Men:Euc.

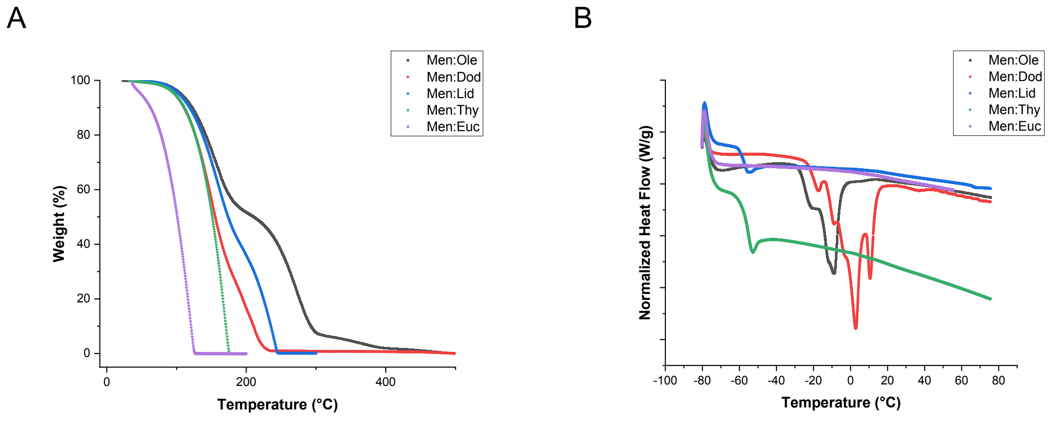

Figure S3. Thermogravimetric analysis (A) and Differential Scanning Calorimetry (B) analysis of the pure HES.

2. Synthesis and characterization of the polymers

2.1. Synthesis of the latexes

Table S1 and **Table S2** present the formulation used for the polymer latexes and the HES containing latex, respectively.

Table S1. Formulation used for the synthesis of the polymers by miniemulsion polymerization.

Reactant	Amount (g)	
	**Homopolymer latex**	**Copolymer latex**
Monomer	40.0	40.0
Water	160.0	160.0
Surfactant	1.8 (2% wbm)	1.8 (2% wbm)
Initiator	0.2 (0.5% wbm)	0.8 (2% wbm)

Table S2. Formulation used for the synthesis of the HES containing latex by miniemulsion polymerization.

Reactant	Amount (g)	
	**Homopolymer + HES latex**	**Copolymer + HES latex**
Monomer	45.0	45.0
HES	45.0	19.3
Water	210.0	243.9
Surfactant	2.0 (2% wbm)	2.0 (2% wbm)
Initiator	0.9 (2% wbm)	0.9 (2% wbm)

2.2. *Characterization of the latexes*

Immediately after synthesis, small samples of each latex—both with and without HES—were taken to determine solid content, monomer conversion, and particle size. For the HES-containing latexes, conversion could not be calculated accurately, as the HES does not evaporate along with water, interfering with the gravimetric determination. The results are summarized in Table S3. Additionally, in Figure S4 are represented the TGA results of both polymers without HES, and the DSC plot of the copolymer.

Table S3. Particle size, solid content, conversion and weight average molar mass of the latexes.

Latex	d_p (nm)	S.C. (%)	x (%)	M_W (kg/mol)
Homopolymer	82	20	95	165
Copolymer	217	20	93	149
Homopolymer+HES	127	22	-	53
Copolymer+HES	124	15	-	103

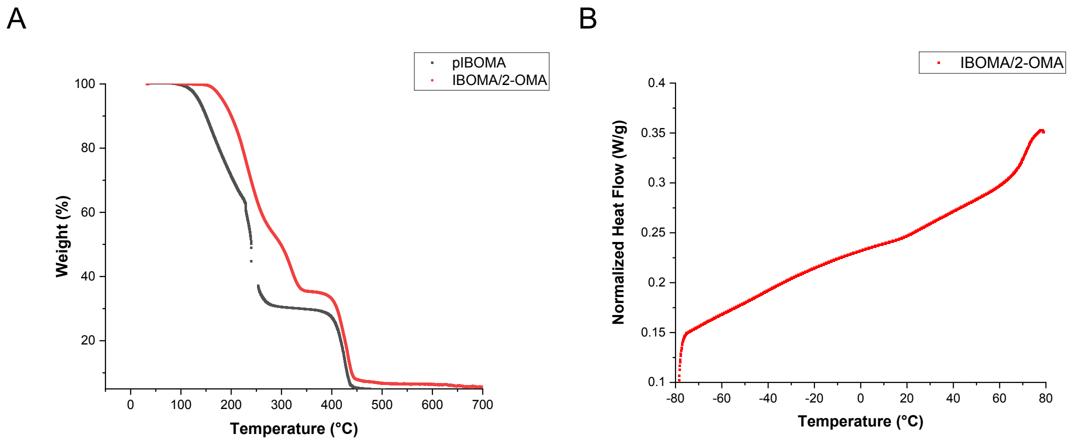

Figure S4. A) Thermogravimetric analysis of the synthesized polymers, B) Differential Scanning Calorimetry of the copolymer.

3. Preparation of the HES-plasticized acrylic films

The evaporation of THF and HESs for the preparation and performance of the acrylic materials was monitored by drying experiments, as presented in Figure S5. As observed, all films reach to an almost plateau after 2-3 h, so this time was taken as the drying time.

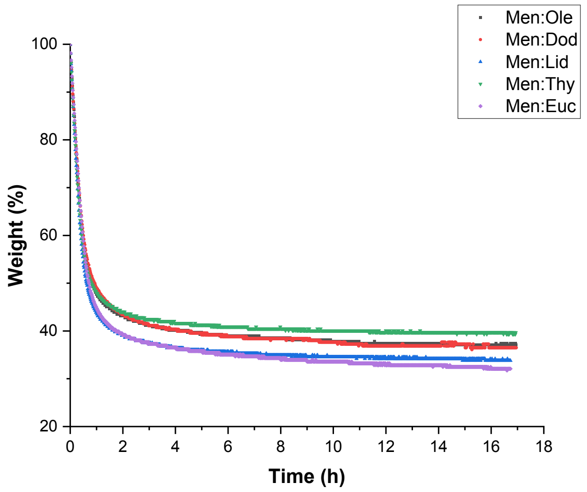

Figure S5. Weight loss of the homopolymers films casted from the THF solution over drying time.

4. Physicochemical properties of the HES-plasticized acrylic films

4.1. Composition of the HES-plasticized acrylic films

The materials were first characterized by FTIR spectroscopy. The spectra of the homopolymer-based films are shown in Figure S6, while those corresponding to the copolymer-based films are presented in Figure S7.

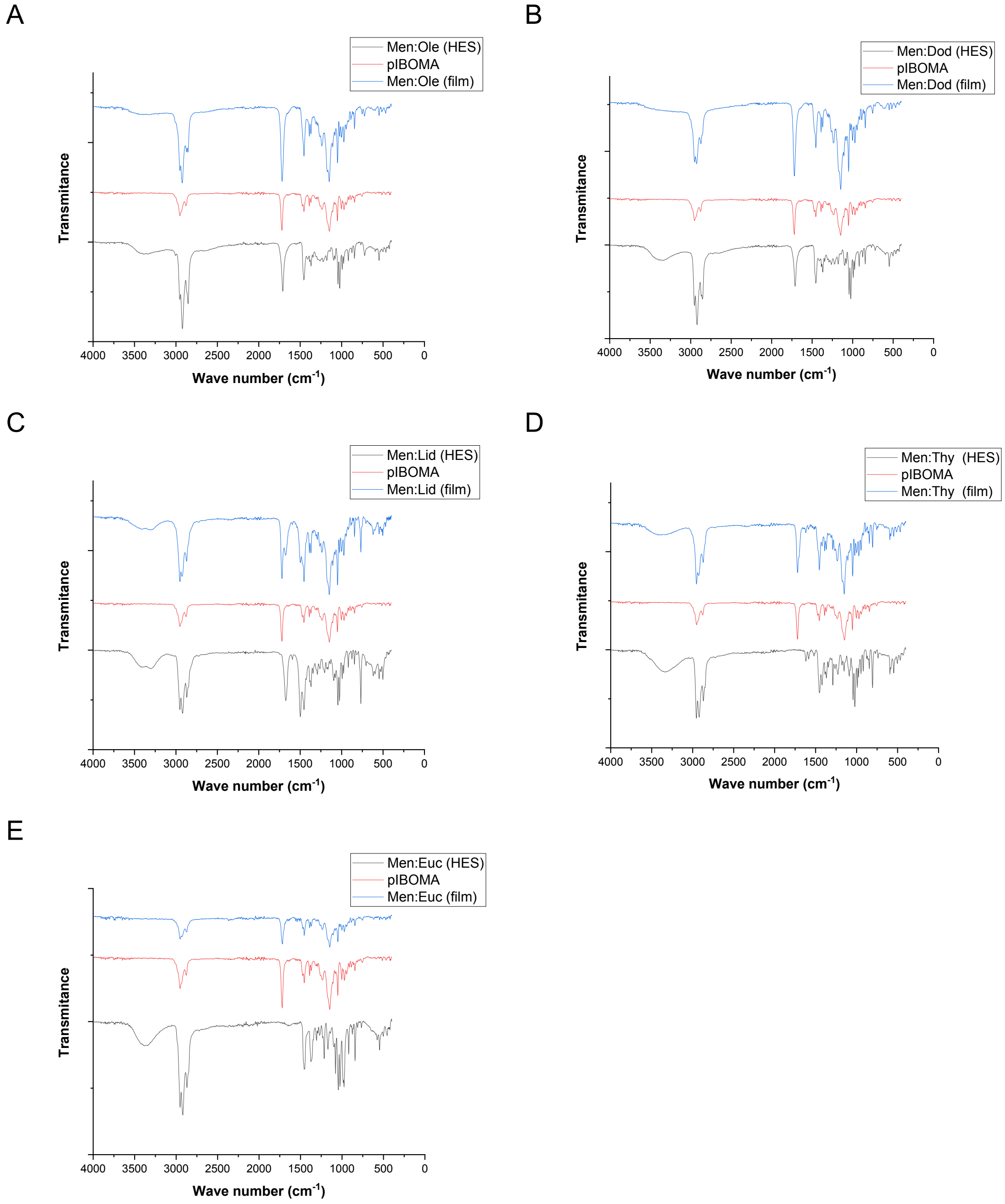

Figure S6. FTIR spectra of the plasticized homopolymer films compared to its components. In black the HES, in red pIBOMA homopolymer and in blue the plasticized film. A) Men:Ole, B) Men:Dod, C) Men:Lid, D) Men:Thy and E) Men:Euc.

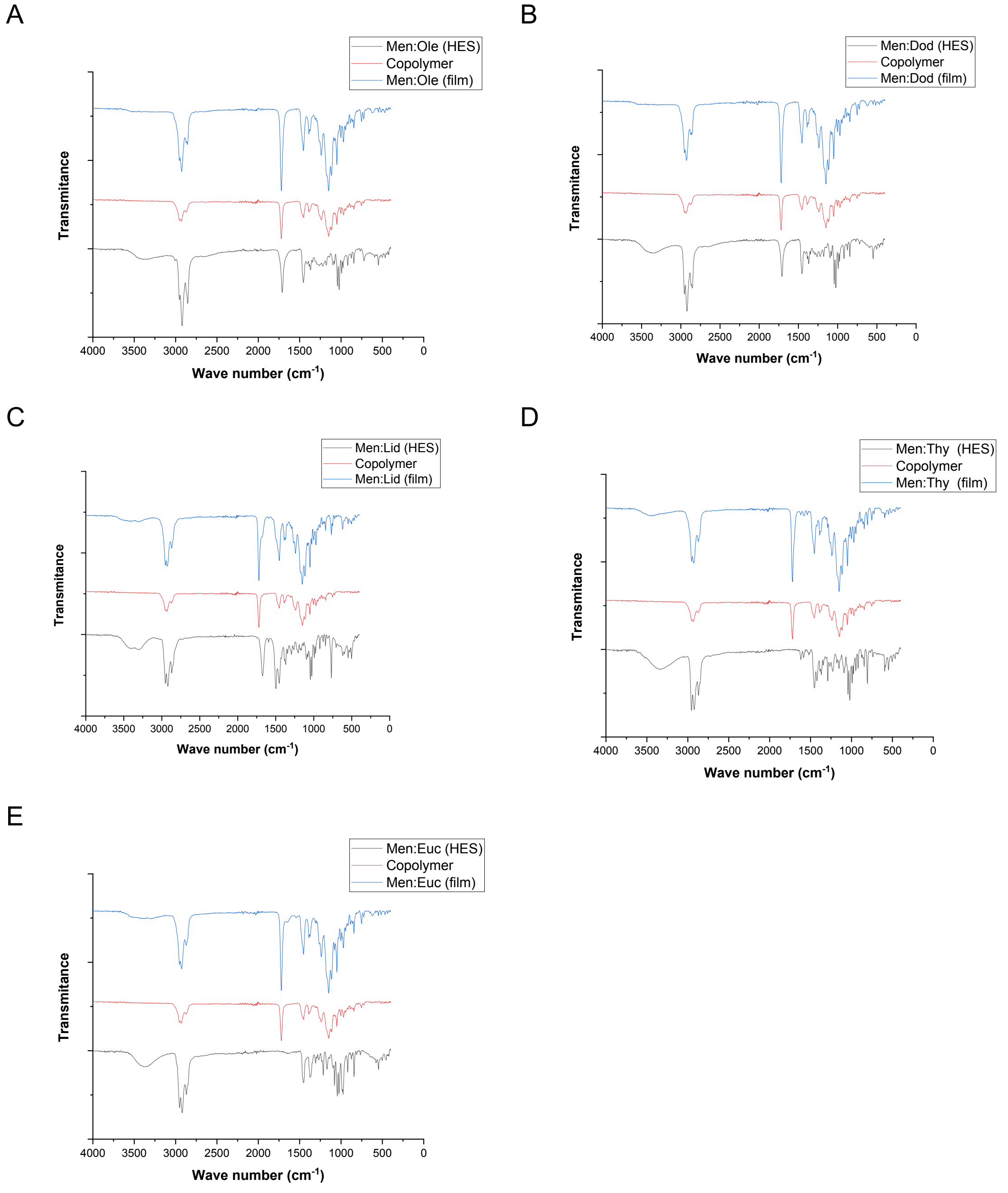

Figure S7. FTIR spectra of the plasticized copolymer films compared to its components. In black the HES, in red IBOMA/2-OMA copolymer and in blue the plasticized film. A) Men:Ole, B) Men:Dod, C) Men:Lid, D) Men:Thy and E) Men:Euc.

4.2. Rheological properties of the HES-plasticized acrylic films

Rheological characterization was performed, and the results are summarized as follows: frequency and temperature sweep data are shown in Figure S8 for the homopolymer films and Figure S9 for the copolymer films. Note that no rheological tests were conducted on the IBOMA homopolymer film containing Men:Euc, as the material was too rigid to be deformed under the applied conditions.

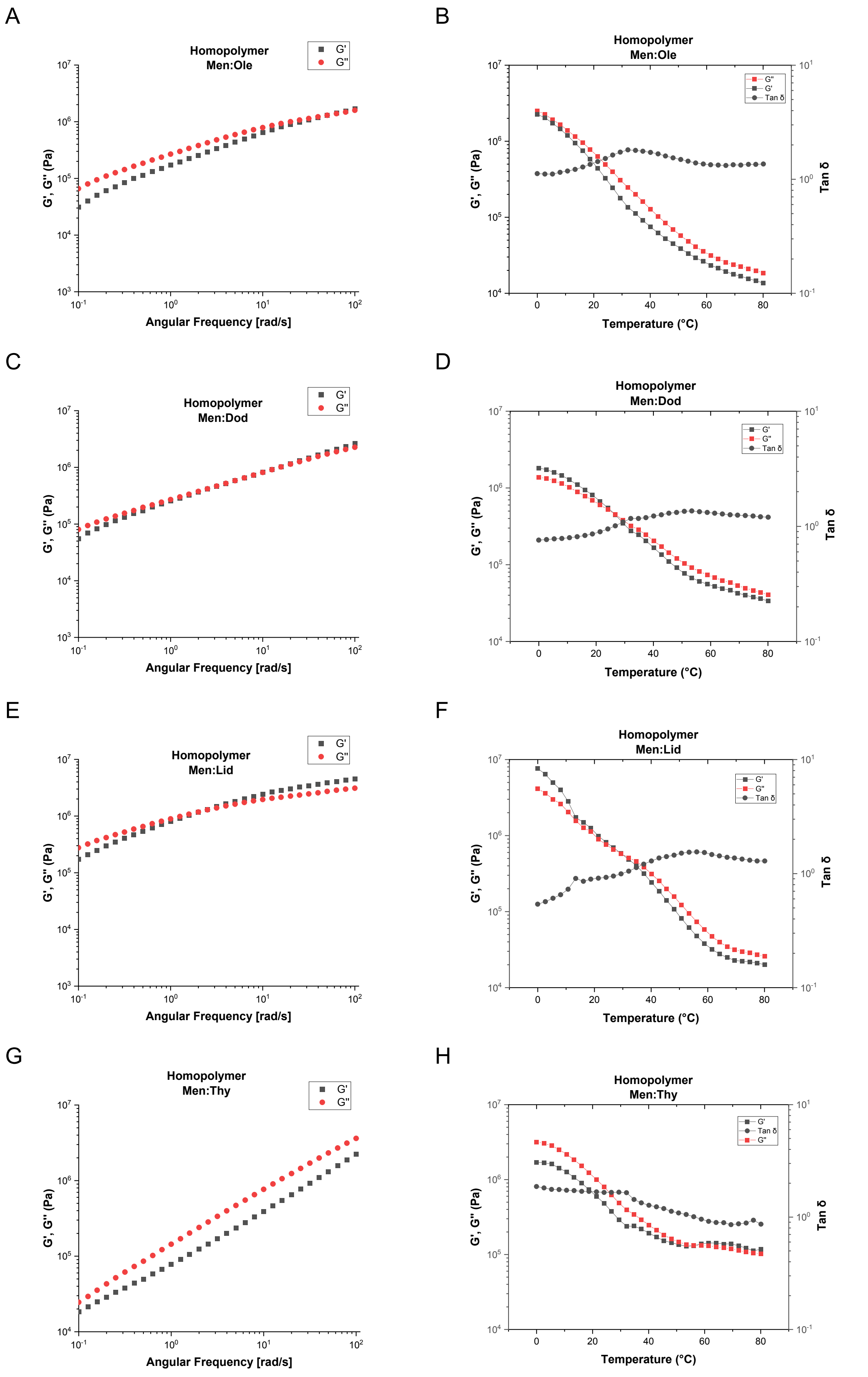

Figure S8. Frequency (left) and temperature (right) sweeps for IBOMA homopolymer plasticized films. A, B) Men:Ole, C, D) Men:Dod, E, F) Men:Lid and G, H) Men:Thy. Men:Euc could not be measured.

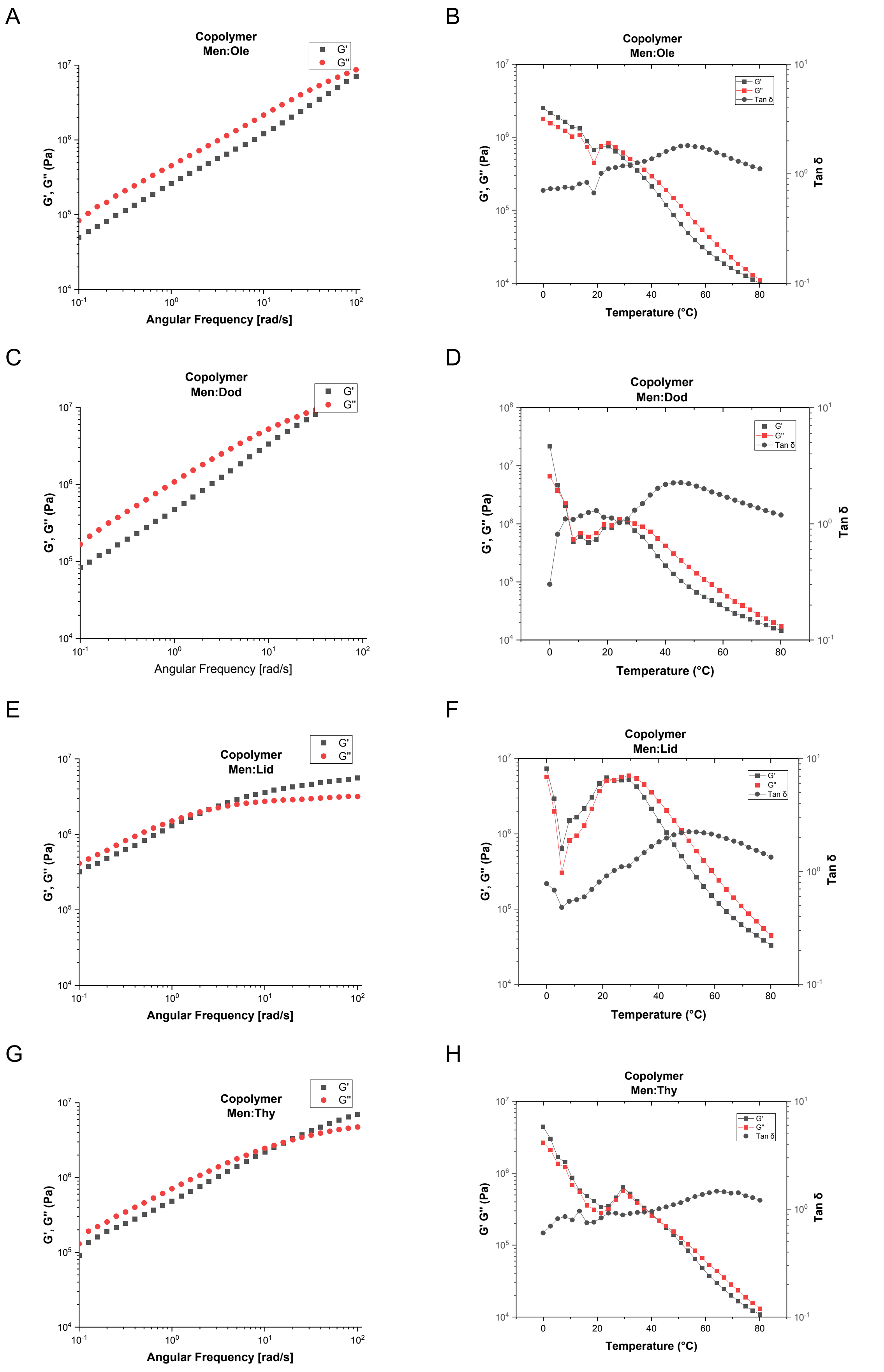
A
Copolymer
Men:Ole
G'
G''
G', G'' (Pa)
Angular Frequency [rad/s]
B
Copolymer
Men:Ole
G'
G''
Tan δ
G', G'' (Pa)
Tan δ
Temperature (°C)
C
Copolymer
Men:Dod
G'
G''
G', G'' (Pa)
Angular Frequency [rad/s]
D
Copolymer
Men:Dod
G'
G''
Tan δ
G', G'' (Pa)
Tan δ
Temperature (°C)
E
Copolymer
Men:Lid
G'
G''
G', G'' (Pa)
Angular Frequency [rad/s]
F
Copolymer
Men:Lid
G'
G''
Tan δ
G', G'' (Pa)
Tan δ
Temperature (°C)
G
Copolymer
Men:Thy
G'
G''
G', G'' (Pa)
Angular Frequency [rad/s]
H
Copolymer
Men:Thy
G'
G''
Tan δ
G' G'' (Pa)
Tan δ
Temperature (°C)

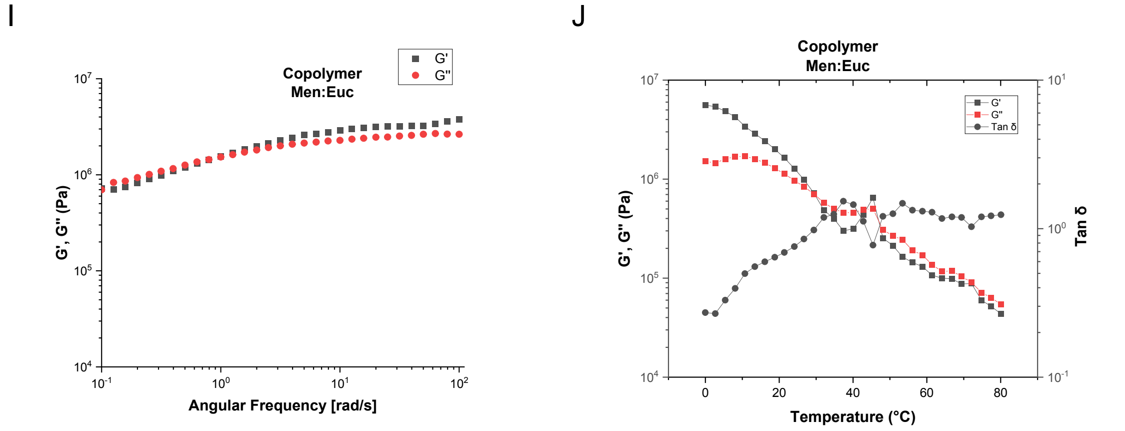

Figure S9. Frequency (left) and temperature (right) sweeps for IBOMA/2-OMA copolymer plasticized films. A, B) Men:Ole, C, D) Men:Dod, E, F) Men:Lid, G, H) Men:Thy and I, J) Men:Euc.

4.3. Potential of the HES-plasticized films as therapeutic patches

Water uptake experiments were carried out to the copolymer films, and the immersion water was analyzed by ^{1}H-NMR. All the spectrums are collected in Figure S10.

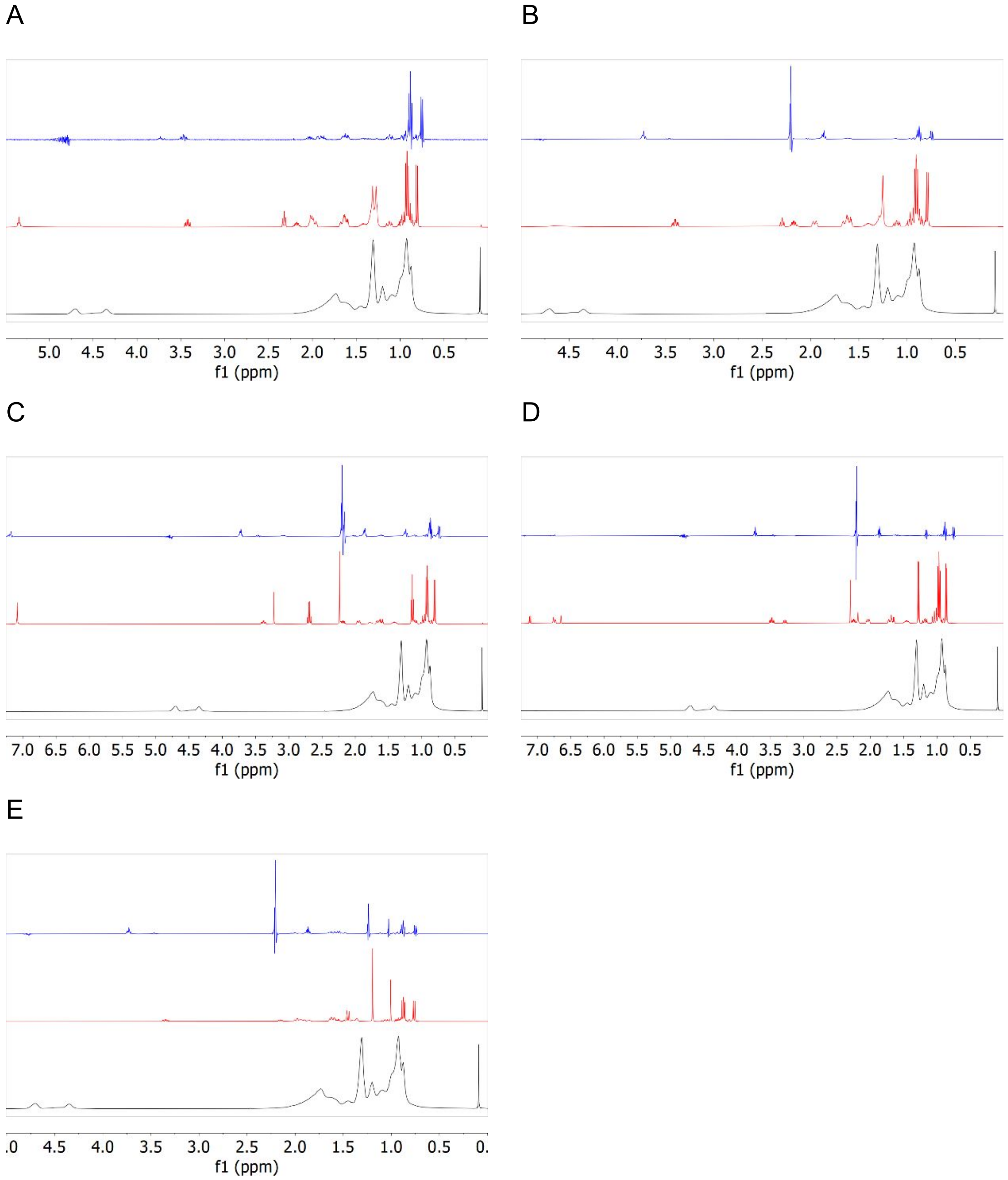

Figure S10. ^{1}H-NMR spectra of the immersion water of plasticized copolymer films' water uptake tests compared to the films' components. In black the IBOMA/2-OMA copolymer, in red the HES and in blue the water. A) Men:Ole, B) Men:Dod, C) Men:Lid, D) Men:Thy and E) Men:Euc.
